# Research on static seating comfort of the Chinese population under different seat angle design parameters

**DOI:** 10.3389/fbioe.2025.1592166

**Published:** 2025-06-13

**Authors:** Lingbo Yan, Lingchun Yu, Na Li, Luozhe Shangguan, Muni Luo

**Affiliations:** ^1^ State Key Laboratory of Advanced Design and Manufacturing for Vehicle Body, Hunan University, Changsha, China; ^2^ Department of Radiology, Xiangya 3rd Hospital, Central South University Changsha, Changsha, China; ^3^ Country Garden School in Ningxiang City, Changsha, China

**Keywords:** seating comfort, Chinese human finite model, body pressure distribution, intervertebral disc stress, vertebral body stress

## Abstract

**Introduction:**

This study aimed to deepen the understanding of seating comfort for the Chinese population by developing a human finite element (FE) model.

**Methods:**

This model was integrated with a specific vehicle seat FE model to construct a comprehensive human-seat FE model, and the mechanical responses of the human body were analyzed under varying seat angles. Body pressure distribution, intervertebral disc stress and strain, and vertebral body stress were examined to study the relationship between the internal reactions of the human body and surface contact conditions.

**Results:**

The results indicate that when the seat is flipped, the trends of disc stress, average pressure, and contact area are consistent, and the maximum strain closely aligns with the maximum pressure. When the backrest is adjusted, lumbar spine stress and surface pressure exhibit similar trends, while disc stress, strain, and the 1-SPD value show consistent patterns.

**Discussion:**

The study concludes that increasing the backrest angle does not necessarily enhance comfort. Moreover, the stress variations in the thoracic and lumbar spines correlate with spinal angle alterations, suggesting that spinal angle can serve as a reliable indicator of stress conditions. Finally, the study highlights the correlation between spinal force and body pressure distribution, underscoring the utility of body pressure distribution metrics as a valuable proxy for understanding spinal responses.

## 1 Introduction

The Chinese human finite element (FE) model is used to study seating comfort, which helps overcome the limitations of the current European and American human body models in analyzing seating comfort and the mechanical response of the human body, particularly in terms of the internal spine.

Among various comfort-related components, the seat plays a pivotal role as it is the primary interface between the human body and the vehicle. Various elements affect static seat comfort, including human sitting posture, seat shape, material properties, and the inclination angles of the seat cushion and backrest (Bahrampour et al., 2020; Li et al., 2020; Havelka et al., 2021; Zhang et al., 2021). A comprehensive analysis of these factors is essential for accurately evaluating seat comfort. Such analyses provide valuable insights for optimizing seat design parameters, enabling improvements in comfort, reducing driver fatigue, and enhancing the overall driving experience. In the field of sitting comfort studies, two primary methodological approaches are commonly applied: finite element modeling (FEM) and inverse dynamic analysis using musculoskeletal modeling (Zhong et al., 2025). The FEM approach enables detailed stress distribution analysis, yet it is constrained by its fixed structural framework, which limits its adaptability to varying sitting postures. Conversely, inverse dynamic analysis with musculoskeletal modeling offers greater flexibility by allowing the adjustment of intervertebral disc angles to simulate adaptive spinal configurations across different postures. Liu et al. (2021) performed two-dimensional adjustments of the spine in the sagittal plane to obtain the contact load and interface pressure distribution in the back region under different spinal postures. Zhong et al. (2022) established a posture-adjustable spinal model to study contact pressure in more postures within 3D space. However, this method provides comparatively less granularity in stress analysis.

In the realm of objective evaluation, the pressure distribution at the human–seat contact interface has emerged as a widely adopted and reliable method due to its close correlation with subjective comfort assessments. Mergl et al. (2005) used the method of body pressure distribution to discover the correlation between pressure and discomfort and applied the results to predict long-term driving discomfort. Franz (2010) studied the optimal design of headrests and lumbar support using body pressure distribution (Franz, 2010). Gross et al. (2020) and Ren and Zhang (2023) assessed seat comfort using this method. Zenk et al. (2006) investigated human body pressure distribution under six different backrest and seat cushion conditions and developed a short-term discomfort model specifically for seat cushions and backrests. Lantoine et al. (2021) studied the comfort of seats under long-duration driving conditions using body pressure distribution. Their findings emphasized that the maximum pressure should ideally be located beneath the ischial tuberosity and gradually decrease toward the thighs and sides. A mismatch in body pressure distribution with physiological structures can lead to altered muscle activation forces and joint forces, thereby increasing fatigue.

Although body pressure distribution analysis offers valuable insights into pressure and its distribution at the human–seat interface, its depth in evaluating overall comfort—particularly in the context of biomechanics—remains limited. Key biomechanical aspects, such as muscle load, subcutaneous soft tissue stress, and spinal force, are not fully addressed. To bridge this gap, researchers have increasingly turned to medical technologies to deepen the understanding of biomechanical responses. For instance, Lee et al. utilized X-ray or MRI systems to scan the spines of volunteers in various postures, including different backrest angles, standing, and supine positions. They analyzed trends in spinal curves and segmental angle changes (Lee et al., 2014; Sato et al., 2021). Wilke et al. (2001) and Zenk et al. (2007) implanted pressure sensors into the human body to measure intervertebral disc pressure, identifying optimal seat settings that minimize disc pressure. Gao (2017) and Xiong (2021) explored muscle activation by analyzing electromyogram (EMG) signals, providing insights into muscle utilization during sitting. These studies highlight the importance of integrating body pressure distribution data with biomechanical indicators to achieve a more comprehensive and accurate evaluation of seat comfort.

Physiological responses such as the curvature of the spine, stress on vertebral bodies and intervertebral discs, and muscle forces offer objective indicators for evaluating comfort. However, these physiological responses are limited by challenges in measurement, difficulty in obtaining data, and the invasive nature of their assessment. High-precision human finite element models provide a solution by enabling finite element simulations that not only capture body pressure distribution characteristics but also offer insights into biomechanical responses, including muscle forces, internal tissue stress, and spinal forces—parameters that are difficult to measure directly in experiments (Kim et al., 2010; Gao et al., 2023). Extensive research has been conducted by scholars worldwide using detailed human models to analyze human–seat interactions. For instance, Xu et al. utilized CT scan data to develop a finite element model of the L4–L5 lumbar spine and intervertebral disc segments, and their study revealed a stress concentration on the posterior and posterolateral sides of the intervertebral disc in a sitting position (Xu et al., 2023; Dong et al., 2024). Ye et al. established a human–seat comfort simulation model and validated the feasibility of finite element simulations for evaluating and optimizing seat comfort by comparing simulation results with experimental body pressure distribution data (Ye et al., 2016; Tong et al., 2022). Similarly, Hanumantha and Smith optimized the THUMS human finite element model to create the SAFER HBM model. They conducted simulations on two seat models (sedan and SUV) with four different foam hardness levels and proposed an objective method for predicting seat comfort (Hanumantha and Smith, 2022). Siefert et al. used the Casimir model to study the dynamic comfort of the human body (Siefert and Hofmann, 2019). By constructing human and seat finite element models and performing simulation calculations, these studies have successfully replicated human–seat interactions, thereby accelerating the seat design process while reducing development time and costs.

The purpose of this paper is to utilize a Chinese-specific human–seat finite element model to explore the variations in human riding comfort under different backrest inclination angles and whole-chair flipping angles. The main work includes establishing a Chinese human finite element model and combining it with the seat model for verification. The seat angle parameters that affect the static comfort of the seat were studied, and the human–chair finite element simulation working conditions under different seat angles were constructed. The comfort of the seat was evaluated by comparing and analyzing the body pressure distribution, the angle of the spine segments, and the stress of the vertebral body and intervertebral discs.

## 2 Methods

### 2.1 Establishment and verification of the human body model

**FIGURE 1:**
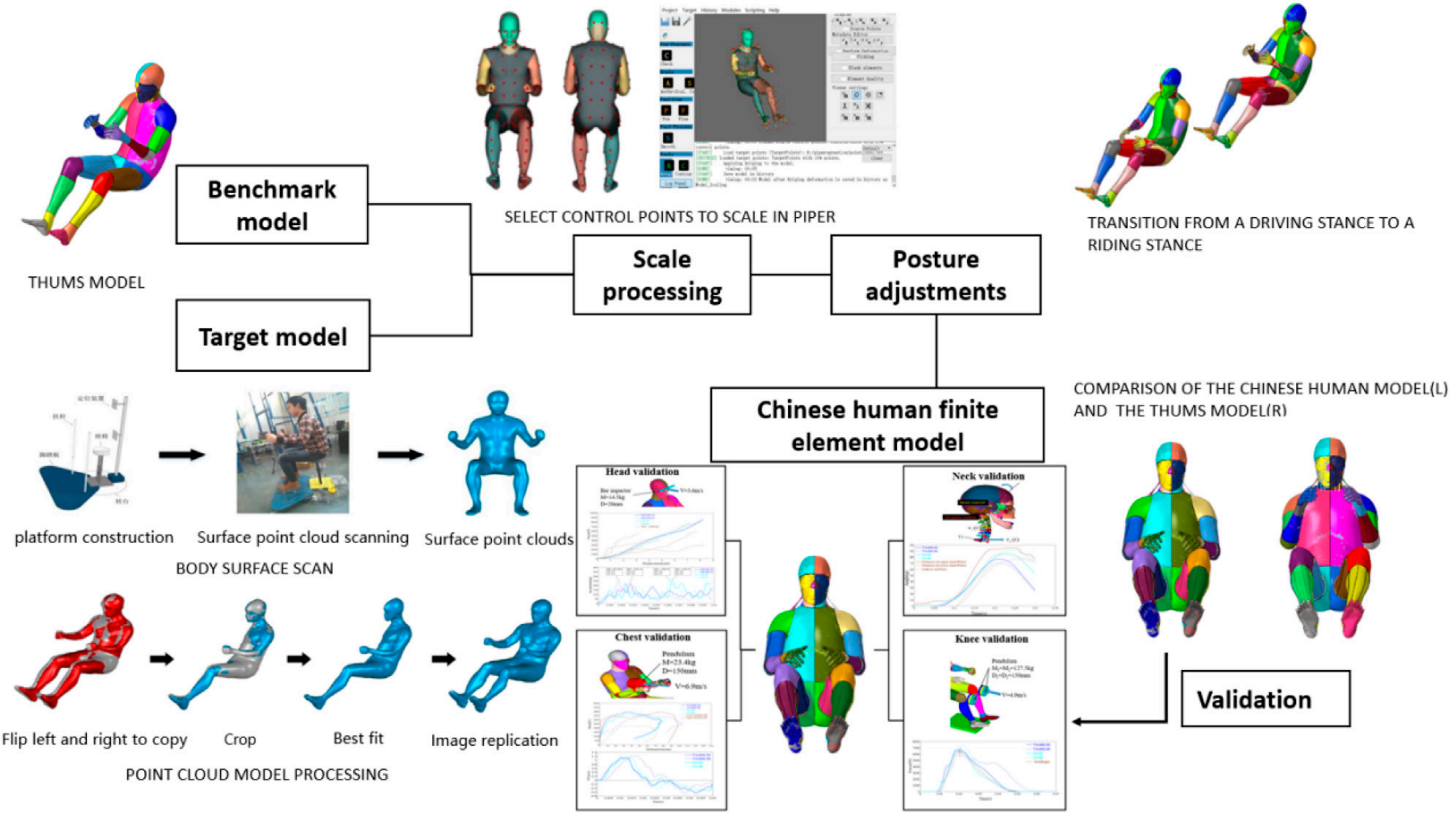
Establishment and verification of the Chinese human finite element model.

The Chinese human finite element model used in this study was developed by scaling the THUMS AM50 human model. Due to its extensive application in human injury prediction simulations, it serves as an ideal reference model. Given the structural similarities across human bodies, using the THUMS AM50 model as a base ensures that the scaled Chinese finite element model retains sufficient biological accuracy. To develop the Chinese human finite element model, a body-surface point-cloud model of a 50th-percentile Chinese male population was obtained by scanning the outer contours of four male volunteers whose body dimensions matched the statistical data for the 50th-percentile Chinese population. The body surface models of four volunteers were averaged to establish the most representative 50th-percentile Chinese body surface model: one of the four body surface models was selected as the base model, while the remaining three were used as the target models; a total of 69 landmark points were selected on the base and target models, and one-to-one correspondence between the target model point cloud and the base model point cloud was achieved using radial basis interpolation and feature point matching. The base model matrix is defined as $\bar{X_{g}}$ , and all models are adjusted to the position of the base model by the orthogonal rotation matrix R. The minimum value of $\left\|X_{g}R-\bar{X_{g}}\right\|$ is calculated using the dinorm of the solution matrix so that the difference between all models and the average model is minimized. The matrix R can be obtained using MATLAB. The shape of all matrices is adjusted to the average matrix by rotating the matrix, and the average shape of the four individual surface models can be obtained after many iterations of adjustment. The final point-cloud model was used as the target for scaling the THUMS human model. The scaling process was conducted using PIPER software with the Kriging module, which enabled precise adjustments to align the THUMS model with Chinese anthropometric characteristics. As shown in Figure 1, 142 symmetrical body surface landmarks were selected as the control points for grid scaling, which covers the key features of the body surface, such as the nose bridge point, the top of the head, the acromion point, the fingertip point, the sternum point, the knee-joint point, and the ankle-joint point. A number of control points on the original finite element model were selected, and then, an equal number of target points were taken at the same position on the target geometry surface. The execution of the deformation control was determined, and software automatically started smoothly scaling the model from the control points to the target points. To ensure that the scaled model accurately simulates the mechanical response characteristics of the human body, it underwent an impact test to verify its accuracy and reliability. Additionally, using the keyword *BOUNDARY_PRESCRIBED_MOTION to apply a forced displacement to the FE model, the model’s posture was adjusted from the driving posture to an alternate posture to accommodate subsequent simulation analyses. Figure 1 illustrates the process of constructing and validating the Chinese human finite element model. Detailed information on body-surface scanning and model-scaling configurations can be found in the previous work of the research team (Zhang, 2021; Ye, 2022).

### 2.2 Establishment of the seat model

**FIGURE 2:**
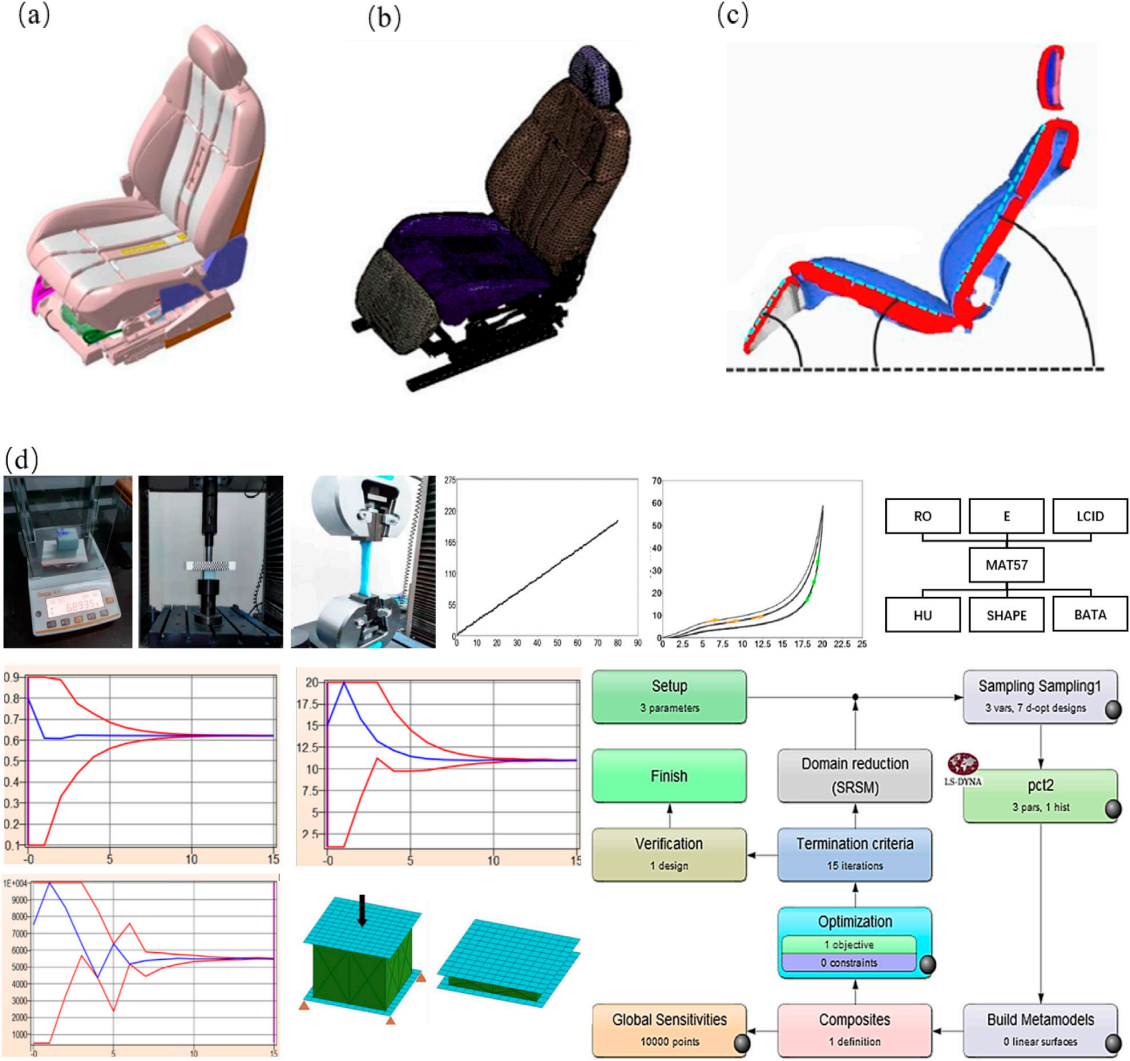
Establishment of the seat finite element model. **(a)** CAD model of the seat, **(b)** finite element model of the seat, **(c)** sectional view of the simplified seat, and **(d)** determination of foam material parameters.

As illustrated in Figures 2a,b, the CAD geometric model of the seat comprises five main components, namely, the seat frame, foam, embedded steel wire, seat pan, and suspension spring. This CAD model was further subdivided into a finite element model using ETA/PreSys software. The FE model of the seat has been simplified to keep only the seat cushions, backrest, headrests, and leg rests, and the sectional view is shown in Figure 2c. The material properties and mesh sizes adopted in the model are summarized in Table 1. For the foam material, MAT57 was selected due to its suitability for modeling nonlinear viscoelastic materials. Key material parameters for MAT57 include density (RO), tensile elastic modulus (E), compressive load curve (LCID), hysteretic unloading coefficient (HU), unloading shape factor (SHAPE), and creep decay constant (BETA). Tensile and compression tests were performed to obtain essential parameters such as density, tensile elastic modulus, and compressive load curve. These experimental results were then used to refine the foam material model. To determine the optimal values for parameters such as HU, SHAPE, and BETA, LS-OPT software was used for parameter simulation and optimization. The optimization process aimed to enhance the accuracy of the foam material’s behavior under varying conditions. A schematic representation of the parameter optimization process is shown in Figure 2c. Detailed descriptions of the optimization procedure and its implementation can be found in the previous study by Zhang (2021).

**TABLE 1:** Seat finite element material parameters.

Parameters	Frame	Foam	Embedded steel wire	Sitting basin	Suspension spring
Element type	Shell element	Tetrahedral element	Beam element	Shell element	Beam element
Material property	MAT24	MAT57	MAT1	MAT24	MAT1
Grid size	10 mm	8 mm	10 mm	10 mm	10 mm

### 2.3 Establishment and verification of the human–seat finite element model

**FIGURE 3:**
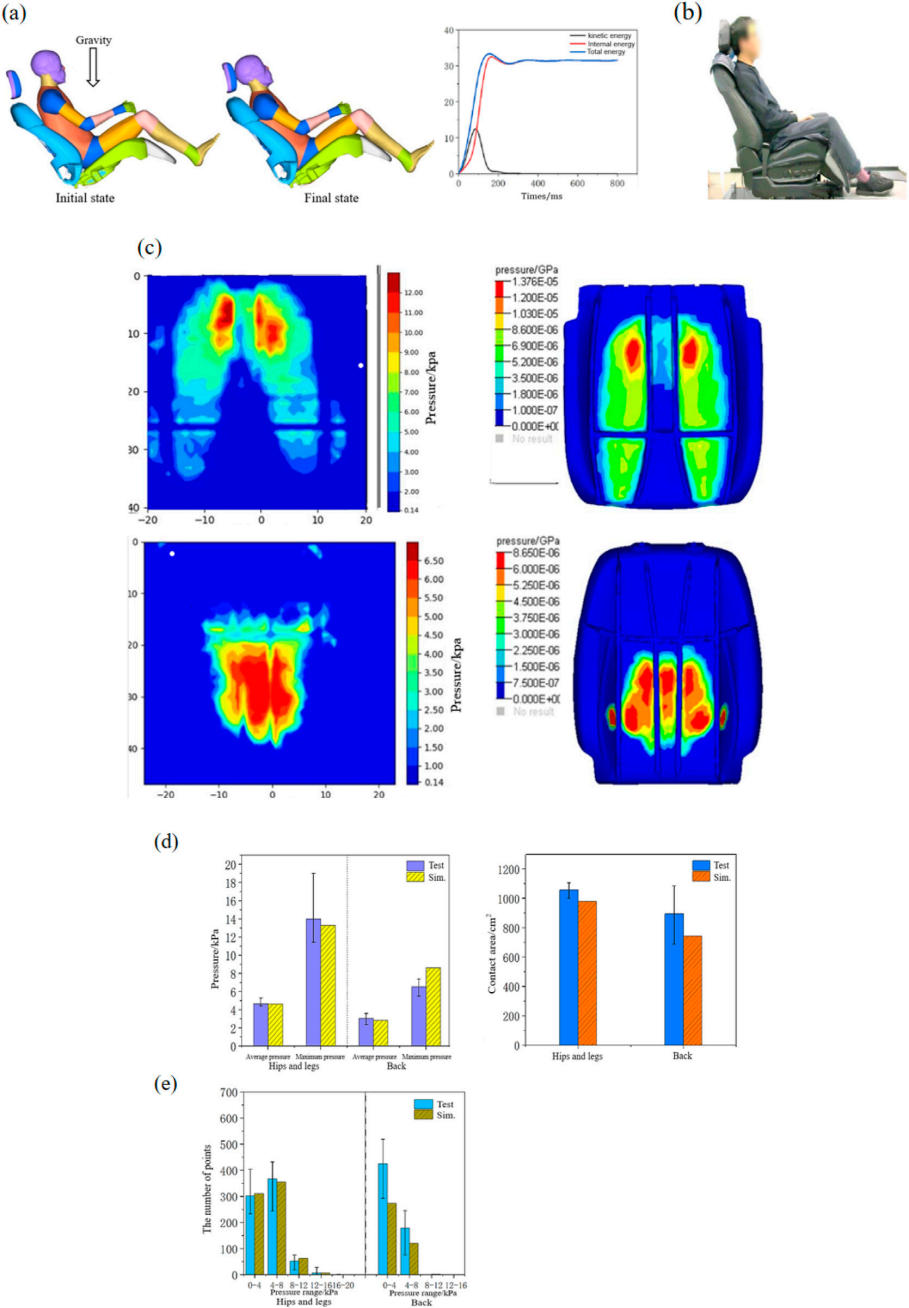
Comparison of the simulation and experiment. **(a)** Simulation model settings and the energy changes, **(b)** body pressure distribution test, **(c)** comparison of body pressure distribution contours, **(d)** comparison of the simulation and experiment values of the body pressure distribution, and **(e)** comparison of the number of pressure points.

Coupling the human model and the seat model: first, the rotation and movement tools were used to bring the human body close to the seat, keeping a distance of 5–10 mm; second, the four contact surfaces—between the human hips and legs and the seat cushion, the waist back and the backrest, the head and the headrest, and the calves and the leg rest—were automatically used to contact the keyword *CONTACT_AUTOMATIC_SURFACE_TO_SURFACE, and the friction coefficient was 0.3 (Derler and Gerhardt, 2012). Finally, the *LOAD_BODY_Z keyword was used to apply a gravity field of 9.816 N/kg to the model to make the human body fall onto the seat, while the hands and feet were not restrained in any way, and the joints were not locked, allowing the human body to sit naturally in the seat. The simulation time was set to 1.2 s to ensure that it was long enough for the human body to sit stably. The simulation model settings and the energy changes during the model fall simulation are shown in Figure 3a. By analyzing the energy changes during the model fall simulation, it was found that during the falling process of the human body, the energy gradually increased from 0 to 100 ms. Due to the compression deformation of the foam and the human body, the internal energy also increased. After 400 ms, the energy reached equilibrium, and there was basically no change in the relative position of the human body with respect to the seat. The dynamic effects of the seat were not taken into account.

The human–chair finite element model is verified by comparing the body pressure distribution results obtained from the simulation with those obtained from the experiment. Four 50th-percentile male volunteers with a height of 168 ± 8 cm and a weight of 69 ± 6 kg were selected for body pressure distribution testing, as shown in Figure 3b. Body pressure distribution tests use capacitive pressure distribution sensors from XSENSOR, Inc.; volunteers sat in the seat with body pressure distribution sensors and remained stationary. Professional 7.0 software was used to obtain the average pressure, maximum pressure, and contact area parameters. The human–chair finite element model was used to simulate under the same conditions, and the calculated simulation values were compared with the experimental values. Figure 3c shows the comparison of the pressure distribution, and the results of the experiment and simulation are basically the same: the maximum pressure of the seat cushion appears at the ischial tuberosity and gradually decreases along the thigh, while the pressure on the backrest is concentrated in the middle of the waist and gradually decreases to the periphery. Figure 3d shows the comparison between the maximum pressure, average pressure, and contact area values, and the average pressure and maximum pressure of the hip and leg obtained from the simulation are consistent with those in the test results. The average pressure of the back is basically consistent with that in the test result, and the maximum pressure of the back is larger than that in the test result, with a percent deviation of 20%. The simulation result of the contact area of the hip and leg is slightly smaller than the test result, and the percent deviation is 2%. Figure 3e divides all compression points into ranges with a pressure of 4 kPa and counts the number of compression points in each range, and the simulation results of hips, legs, and back are basically consistent with the test results.

### 2.4 Simulation scenario setup and output

The mechanism for transitioning the seat angle from a sitting position to a lying position involves two main movements: the overall backward rotation of the entire seat around the recliner’s center and the independent adjustment of the backrest angle to achieve the desired position. Based on this mechanism, seat angle adjustments are categorized into two operational conditions: (1) the overall backward rotation of the seat and (2) the adjustment of the backrest angle. Under the condition involving the overall backward rotation of the seat, five distinct adjustment angles were configured. Simulation calculations were performed to analyze the surface pressure distribution and the internal response of the spine. By exporting simulation data on body pressure distribution, key parameters such as maximum pressure, average pressure, contact area, and seat pressure distribution (SPD) uniformity were calculated. Among them, the formula for calculating SPD is as follows:

$$SPD=\frac{\sum_{i=0}^{n}{\left(p_{i}-p_{ave}\right)}^{2}}{4np_{ave}^{2}},$$

where n is the number of non-zero units of the pressure distribution test pad, $p_{i}$ is the pressure value of each pressure test unit, and $p_{ave}$ is the average pressure. SPD serves as a metric for evaluating the uniformity of pressure distribution under static conditions. A lower SPD value reflects greater pressure uniformity, which is positively correlated with enhanced perceived comfort (Milivojevich et al., 2000). Therefore, a reduction in the SPD value signifies an improvement in seat comfort.

**FIGURE 4:**
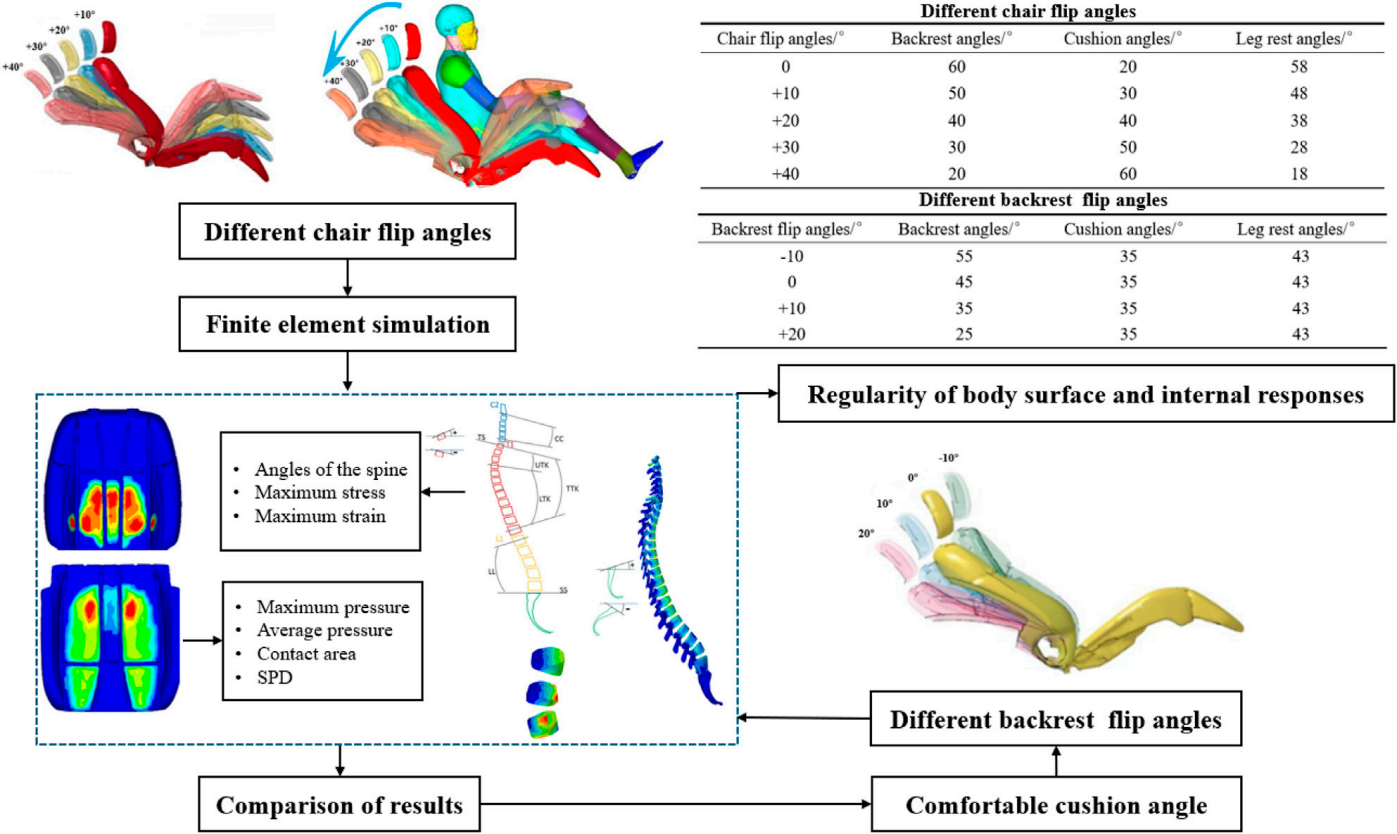
Simulation scenario setup and output.

Internal response metrics include spinal angle, vertebral body stress, and intervertebral disc stress. The spinal angles assessed include cervical lordosis (CC), thoracic kyphosis (TTK), and lumbar lordosis (LL), with specific angle definitions shown in Figure 4 (Sato et al., 2021). By comparing these metrics, the optimal seat cushion angle for comfort was determined, forming the basis for subsequent backrest angle adjustments. Under the condition involving backrest rotation, four different adjustment angles were established. Similarly, the surface pressure distribution and spine response indicators were analyzed to evaluate the impact of the seat angle on static comfort. Specific settings for these conditions and the output indicators are illustrated in Figure 4. LS-DYNA software was used to calculate the simulation, and HyperWorks was used to analyze the results.

## 3 Results

### 3.1 Influence of different chair flip angles

#### 3.1.1 Body pressure distribution results

**FIGURE 5:**
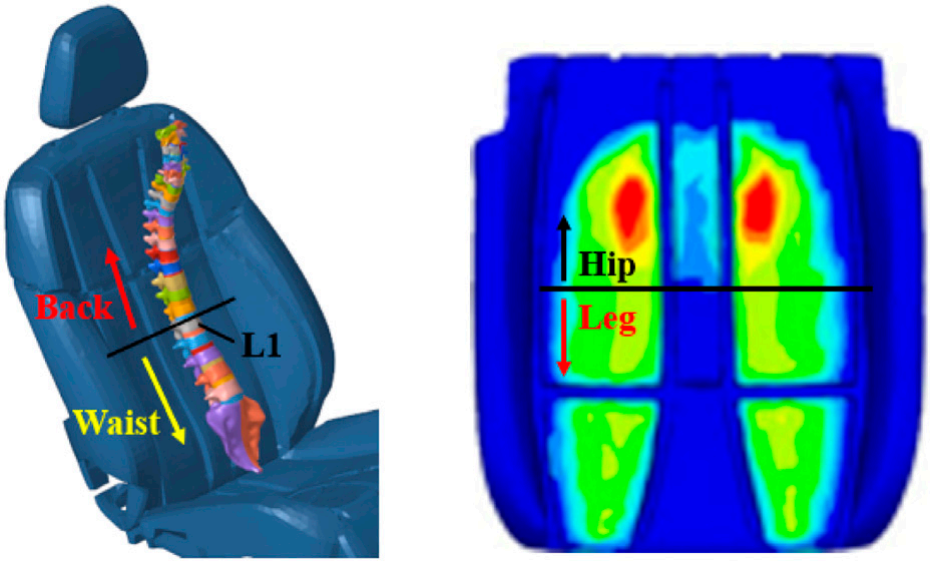
Divisions of the human hip–legs and waist–back.

The divisions of the human hip–legs and waist–back are shown in Figure 5. The waist and back are divided by the projection of the upper end of the L1 vertebral body on the backrest, with the lower part being the waist and the upward part being the back. The hip and legs can be observed from the cushion results.

**FIGURE 6:**
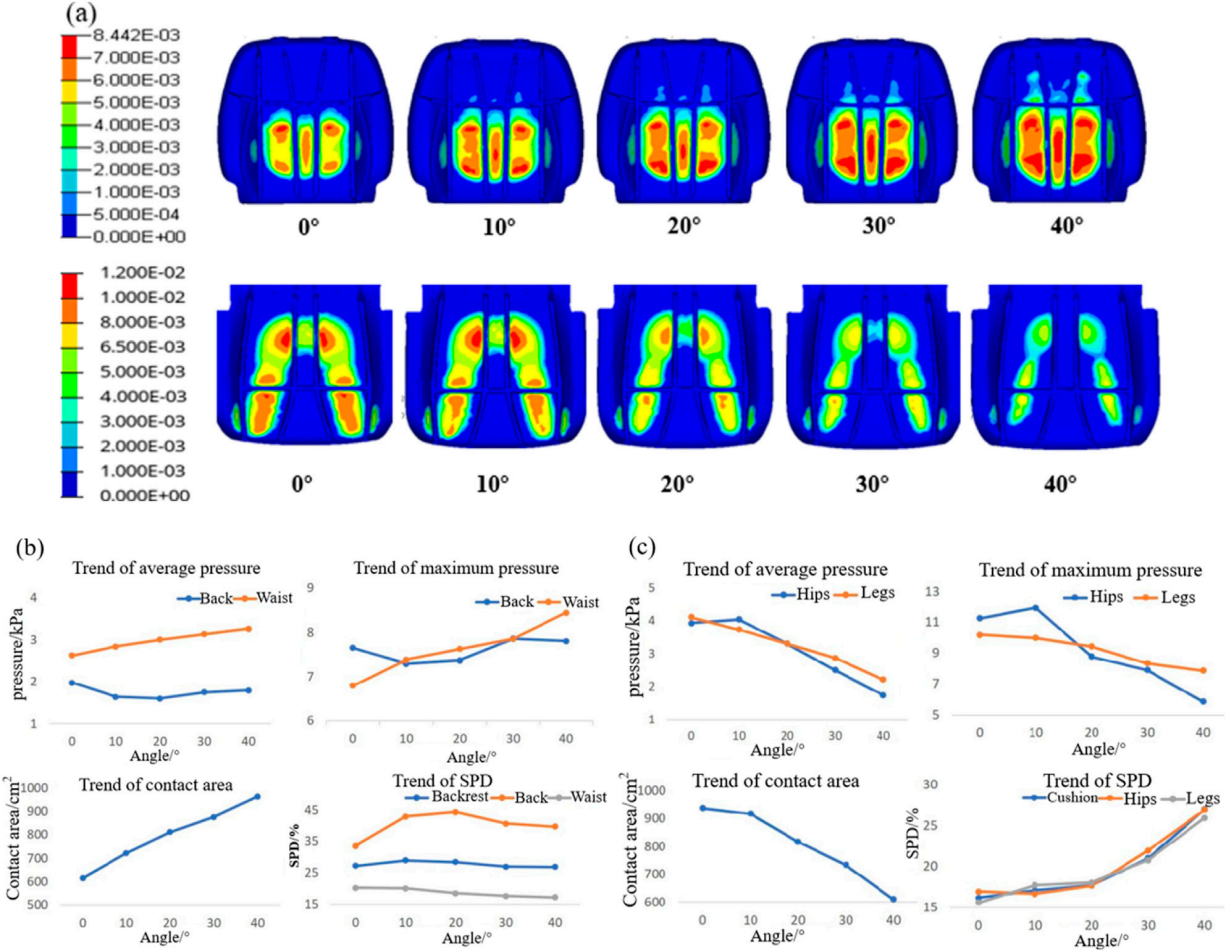
Results of body pressure distribution at different chair-flipping angles. **(a)** Body pressure distribution contour; **(b)** trend of the body pressure distribution index in the waist and back; and **(c)** trend of the body pressure distribution index in the hip and legs.

Figure 6a depicts the pressure distribution at the contact interface between the human body and the seat under varying whole-chair rotation angles. With the backward rotation of the seat, the pressure beneath the human ischial tuberosity gradually diminishes, leading to a reduction in the contact area between the buttocks, legs, and seat cushion. Concurrently, the overall pressure on the back increases, with the contact area extending progressively toward the shoulders. Figures 6b,c show the results of this analysis. The contact area between the human body and the backrest increases as the seat rotates, resulting in an upward trend in both average and maximum pressure on the waist. For the back, however, the average and maximum pressures first decrease and then increase, reaching their lowest values at rotation angles between 10° and 20°. Regarding SPD, the waist consistently demonstrates better pressure uniformity than the back.

As the seat rotation angle increases, the average pressure, maximum pressure, and contact area between the buttocks, legs, and seat cushion exhibit a decreasing trend. At a rotation angle of 20°, the pressure exerted on the legs surpasses that on the buttocks. Simultaneously, the SPD value for the buttocks and legs increases, with a notable increase when the rotation angle exceeds 20°. This indicates that higher rotation angles lead to a more uneven pressure distribution.

#### 3.1.2 Spinal response results

**FIGURE 7:**
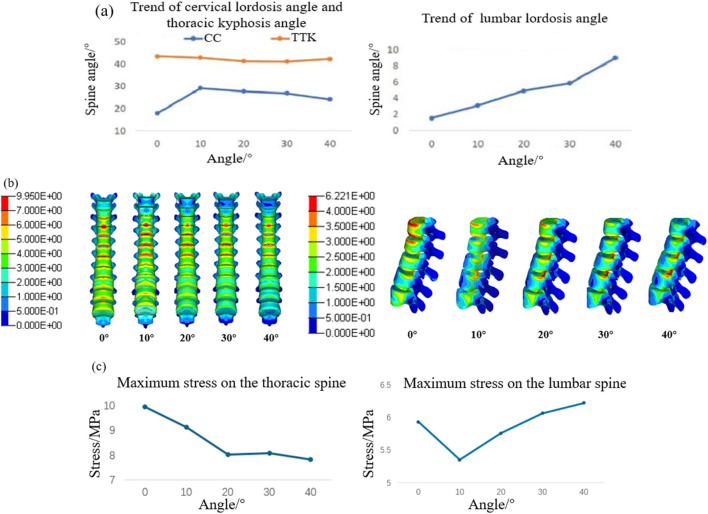
Vertebral response results at different chair flip angles. **(a)** Results of cervical lordosis, thoracic kyphosis, and lumbar lordosis; **(b)** vertebral stress contour; and **(c)** trend of maximum stress on the spine.

The results of the spinal angles under different whole-chair turning conditions are presented in Figure 7a. Across various turning angles, the cervical lordosis and thoracic kyphosis angles remain within the normal range. The cervical lordosis angle peaks when the chair is turned at 10°, while the thoracic kyphosis angle decreases as the turning angle increases. Conversely, the lumbar lordosis angle increases with larger turning angles primarily because the lower back becomes more horizontal, enhancing the backrest’s support for the waist and extending the lumbar spine. Figure 7b shows the stress distribution maps of the T2–T12 thoracic vertebrae and L1–L5 lumbar vertebrae. The results indicate that the maximum stress in the thoracic vertebrae consistently occurs in the anterior region of the T4 and T5 vertebrae. In contrast, the maximum stress in the lumbar vertebrae shifts significantly, moving from the anterior region of the L1 vertebra to the posterior regions of the L3 and L4 vertebrae. Figure 7c illustrates the trends in maximum stress for the thoracic and lumbar vertebrae. As the whole-chair turning angle increases, the thoracic vertebrae experience a reduction in maximum stress, while the lumbar vertebrae exhibit an initial decrease in stress, followed by an increase, with the lowest stress observed at a turning angle of 10°.

**FIGURE 8:**
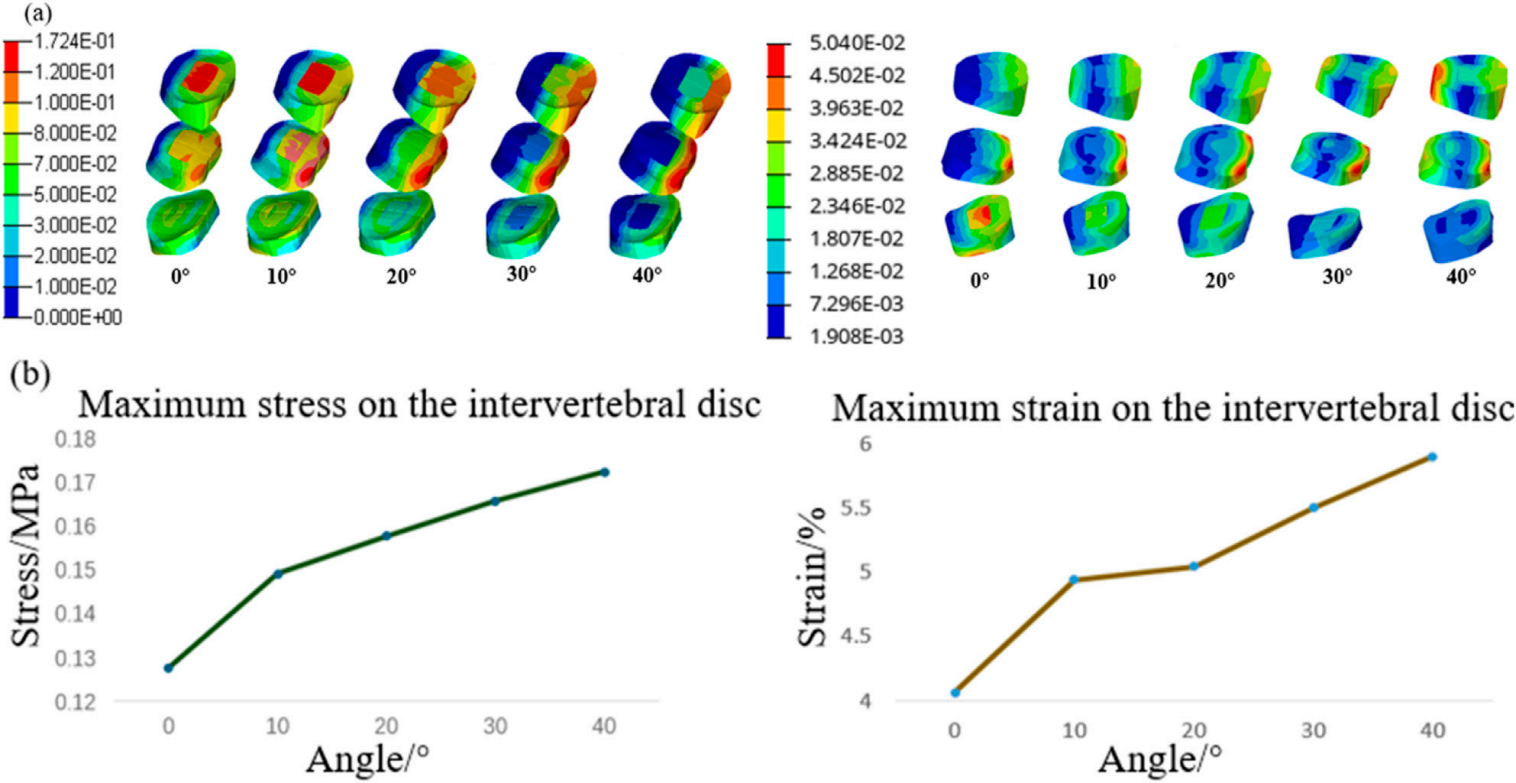
Intervertebral disc response results at different chair flip angles. **(a)** Stress contour (L) and strain contour (R) and **(b)** trend of maximum stress (L) and strain (R).

Since lumbar disc herniation is most common in the L3/L4, L4/L5, and L5/S1 intervertebral discs, these areas were analyzed in detail. Figure 8a depicts the stress–strain distribution maps of these discs. The maximum stress initially appears in the nucleus pulposus of the L3/L4 intervertebral disc and gradually migrates to the posterior annulus fibrosus of the L4/L5 intervertebral disc. Similarly, the maximum strain is initially located in the nucleus pulposus of the L5/S1 intervertebral disc but shifts to the posterior edge of the L4/L5 disc and later to the anterior edge of the L3/L4 disc as the turning angle increases to 40°. Figure 8b shows the trends of maximum stress and strain in the intervertebral discs, both of which increase progressively with larger whole-chair turning angles.

Although the average pressure on the chest and back, along with the stress on the thoracic spine, gradually decreases, the stress on the lumbar vertebral body and the pressure within the intervertebral discs increase significantly, potentially elevating the risk of lumbar pathologies. Given that physiological lesions most commonly occur in the lumbar region, an emphasis on reducing stress in this area is crucial. By comprehensively analyzing the results of body pressure distribution and spinal physiological responses, it is determined that the seat provides optimal comfort when the whole-chair turning angle is maintained between 10° and 20°.

### 3.2 Influence of different backrest flip angles

Building on the conclusions of the previous section, the seat is first rotated as a whole by 15°, after which the effects of varying backrest angles on comfort are studied. The seat cushion angle is held constant throughout the investigation.

#### 3.2.1 Body pressure distribution results

**FIGURE 9:**
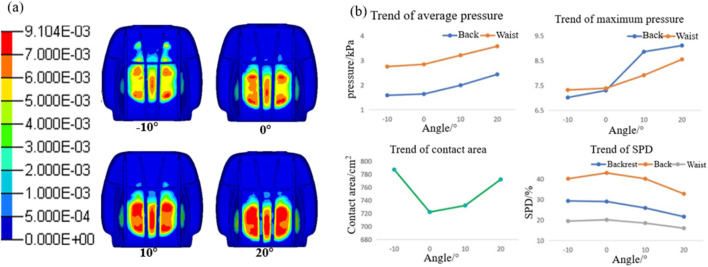
Results of body pressure distribution at different backrest flip angles. **(a)** Body pressure distribution contour of the seat cushion and **(b)** trend of the body pressure distribution index in the waist and back.

The body pressure distribution at the interface between the human body and the seat backrest at varying backrest flipping angles is shown in Figure 9a. As the backrest angle increases, the maximum pressure increases progressively, the contact area shifts downward, shoulder contact decreases, and the support area provided by the backrest changes accordingly. The trends for maximum pressure, average pressure, contact area, and SPD are illustrated in Figure 9b. With an increase in the backrest angle, both the average pressure and maximum pressure increase; however, the pressure distribution becomes more uniform. Notably, the pressure distribution in the waist region is more uniform than that in the back region. The contact area is at its smallest when the backrest flipping angle is 0°. From this baseline, the contact area increases as the backrest is adjusted either forward or backward.

#### 3.2.2 Spinal response results

**FIGURE 10:**
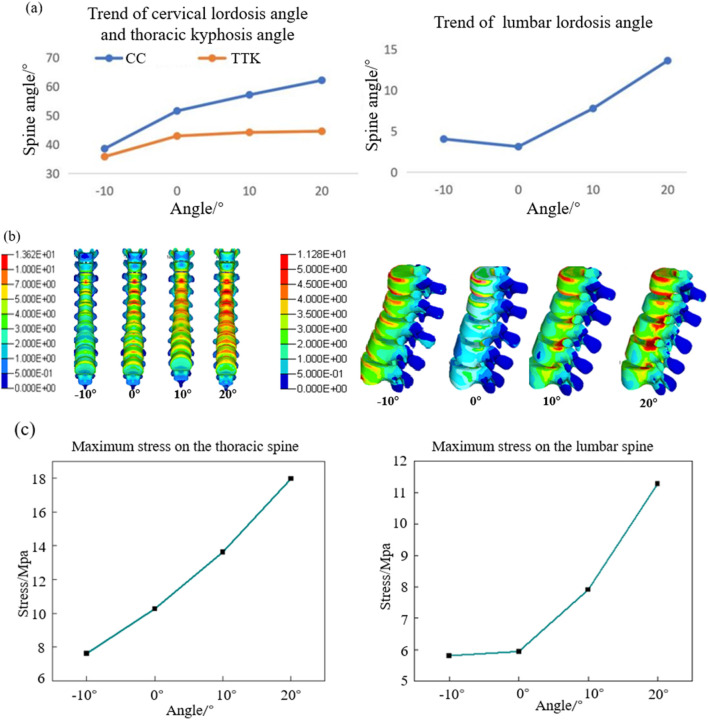
Vertebral response results at different backrest flip angles. **(a)** Results of cervical lordosis, thoracic kyphosis, and lumbar lordosis; **(b)** vertebral stress contour; and **(c)** trend of maximum stress on the spine.

The cervical lordosis angle, thoracic kyphosis angle, and lumbar lordosis angle at different backrest flipping angles are presented in Figure 10a. As the backrest angle increases, both the cervical lordosis and thoracic kyphosis angles increase, with changes in the thoracic spine being less pronounced than those in the cervical spine. The lumbar lordosis angle is at its minimum when the backrest flipping angle is 0°. Figure 10b illustrates the stress distributions of the T2–T12 thoracic vertebrae and L1–L5 lumbar vertebrae at varying backrest angles. Initially, the maximum stress in the thoracic vertebrae is concentrated at the anterior regions of the T4 and T5 vertebral bodies. With an increase in the backrest angle, stress concentration shifts to the superior articular and transverse processes of T2, and the maximum stress eventually localizes at the articular processes of T2. For the lumbar vertebrae, the maximum stress shifts from the anterior region of the L1 vertebral body to the posterior region of the inferior endplate of the L3 vertebral body. As shown in Figure 10c, the maximum stress in both the thoracic and lumbar vertebrae increases as the backrest angle increases.

**FIGURE 11:**
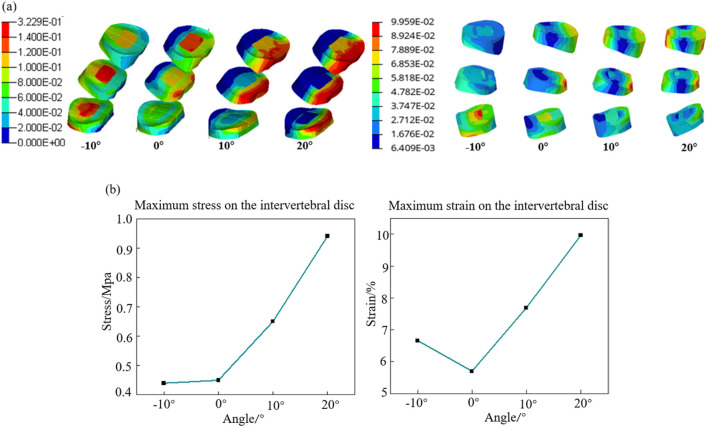
Intervertebral disc response results at different backrest flip angles. **(a)** Intervertebral disc stress contour (L) and strain contour (R) and **(b)** trend of maximum stress (L) and strain(R) on the intervertebral discs.

The stress–strain distributions of the L3/L4, L4/L5, and L5/S1 intervertebral discs are depicted in Figure 11a. When the backrest angle is adjusted forward by 10°, the maximum stress and strain occur in the nucleus pulposus of the L5/S1 intervertebral disc. With no adjustment, the maximum stress is located in the nucleus pulposus of the L3/L4 intervertebral disc, while the maximum strain appears at the posterior-superior edge of the annulus fibrosus of the L4/L5 intervertebral disc. When adjusted backward by 10°, both the maximum stress and strain are concentrated at the posterior-superior edge of the L4/L5 intervertebral disc. At a backward adjustment of 20°, the maximum stress and strain shift to the posterior-inferior edge of the annulus fibrosus of the L3/L4 intervertebral disc. Overall, as the backrest angle increases, the site of maximum stress shifts progressively from the anterior region of the nucleus pulposus to the posterior region of the annulus fibrosus. Additionally, the stress gradually shifts from the lower lumbar discs to the upper lumbar discs. Figure 11b demonstrates that both the maximum stress and maximum strain of the intervertebral discs are at their lowest when the backrest angle is 0°, and they increase steadily within the range of 0°–20°.

## 4 Discussion

Under ideal conditions, the maximum pressure in the buttocks region typically ranges from 7 to 11 kPa, while the maximum pressure in the lower back falls within 4–8 kPa (Kilincsoy et al., 2016; Fan et al., 2022). Analysis of body pressure distribution at different whole-chair turning angles reveals that excessive turning angles result in the maximum pressure in the buttocks falling below the ideal range, while the maximum pressure in the lower back exceeds it. This imbalance indicates insufficient support for the buttocks, which compromises comfort. Therefore, the whole-chair turning angle should be limited to a range of 0°–20° to avoid discomfort in the lower back caused by excessive angles. From a spinal response perspective, the thoracic kyphosis angle and the maximum stress of the thoracic vertebra exhibit a similar trend, both decreasing as the turning angle increases. However, beyond 20°, the rate of change reduces. Conversely, the maximum stress and strain of the lumbar intervertebral disc follow the same trend as the lumbar lordosis angle, increasing with larger angles. This suggests that a greater lumbar lordosis angle intensifies the compressive effect of the posterior edge of the vertebral body on the annulus fibrosus, leading to higher pressure values at the posterior regions of the L3/L4 and L4/L5 annulus fibrosus. Wade et al. (2014) found that intervertebral disc damage typically originates from the posterolateral annulus fibrosus due to its thinner and structurally weaker composition than that of the anterior region, making it more susceptible to injury under stress. Thus, increasing the whole-chair turning angle elevates the risk of intervertebral disc damage. A comfortable whole-chair turning angle should be maintained between 10° and 20°, with the backrest angle ideally set between 40° and 50° and the seat cushion angle between 30° and 40°.

When examining the effect of backrest flipping angles on body pressure distribution, it is observed that both average pressure and maximum pressure increase as the backrest angle increases, indicating improved support and more uniform pressure distribution, particularly in the waist region. However, when the backrest angle exceeds 20°, the maximum pressure in the waist reaches 8.55 kPa, surpassing the ideal range and potentially causing discomfort. Thus, the backrest angle should be limited to a backward adjustment range of 0°–10°. From the perspective of spinal biomechanical responses, stress concentration occurs at the superior articular processes and transverse processes of the thoracic vertebrae. As key structural elements, the articular processes play a vital role in spinal stability and mobility. Excessive backrest angles may increase the risk of thoracic vertebra injury. Consistent with the whole-chair turning conditions, the trends of maximum thoracic vertebra stress align with those of the thoracic kyphosis angle, while the maximum stress and strain of the lumbar intervertebral disc follow the same pattern as the lumbar lordosis angle. This indicates that changes in thoracic kyphosis and lumbar lordosis angles can reflect the maximum stress of the thoracic vertebra and the lumbar intervertebral disc stress and strain. A comprehensive analysis of body pressure distribution and spinal physiological responses shows that a backrest angle within 0°–10° provides the highest comfort.

**FIGURE 12:**
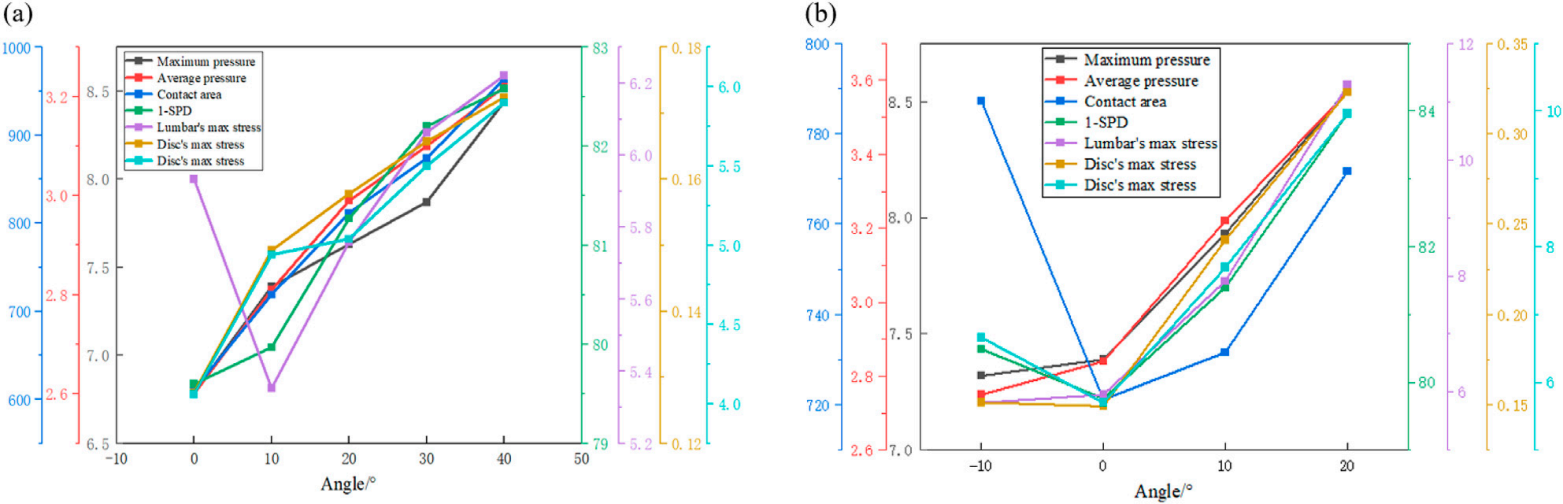
Trend of each index under different seat angles. **(a)** Results of different whole chair flip angles and **(b)** results of different backrest flip angles.

Body pressure distribution is an important method for assessing seat comfort, but it only measures the pressure distribution between the human body and the surface in contact with the seat. In this paper, the surface pressure and the response of the internal spine of the human body are obtained from the simulation, and the relationship between the two is attempted. Figures 12a,b show the results of the maximum pressure, average pressure, contact area, SPD, the maximum stress of the lumbar vertebral body, and the maximum stress and strain of the intervertebral disc at different chair flip angles and backrest flip angles, respectively. Since a smaller SPD value indicates a more comfortable seat, in order to more intuitively reflect the relationship between SPD and comfort, the 1-SPD value is used as a plot indicator; the higher the 1-SPD value, the higher the degree of comfort. Under different chair flip angles, the variation trend of the maximum stress of the intervertebral disc with the average pressure and contact area was the most similar. The trend of maximum strain and maximum pressure is similar. However, none of the four body pressure distribution indexes could reflect the change in the maximum stress of the lumbar vertebrae. Under different backrest flip angles, the maximum stress of the lumbar spine, maximum pressure, and average pressure have similar trends, and the maximum stress and maximum strain of the intervertebral disc have the same trend as the 1-SPD value. In summary, some indicators in the body pressure distribution have the same change law as the spine response under some specific working conditions, and the internal response of the human body can be judged to a certain extent through the results of body pressure distribution. Subsequently, the numerical correspondence between the body surface and *in vivo* indexes can be further explored. At the same time, the angle adjustment interval can be reduced, such as to 5° or even 2°, to further study the optimal seat comfort angle.

The limitations to this study are as follows: (1) only the comfort response of the 50th-percentile Chinese male population was studied, and the results did not reflect the situation of other populations. In the future, parametric modeling should be used to establish more human FE models of different percentiles, BMIs, and ages to study the response characteristics of the Chinese human body. (2) Only the static riding comfort of the human body was considered, and the dynamic response of the human body during the actual driving process was not deeply explored; the established FE model should be used to build a dynamic comfort simulation working condition to study the dynamic riding comfort of the human body. (3) The FE model used lacks the capability for muscle simulation although the influence of muscle force on human comfort is very important. Muscle modeling will be incorporated into this model to further improve comfort research. (4) The verification part only verified the surface pressure distribution of the human body and failed to verify the force of the internal vertebral body and intervertebral discs. Since the model in this paper is scaled from the THUMS model, it can be considered to have similar biological realism to the THUMS model. Subsequently, the spine will undergo multi-condition biomechanical verification, such as tension and compression, as well as flexion and extension, to further validate the effectiveness of the model.

## 5 Conclusion

In this study, a finite element model of a Chinese human–seat interaction was developed to investigate human comfort responses under varying seat angles. This model effectively simulates the contact pressure between the human body and the seat, along with the spinal stresses, providing insights into internal responses that are challenging to measure directly. The findings demonstrate that the seat provides optimal comfort when the whole-chair turning angle is maintained between 10° and 20°, with the backrest angle ideally set between 40° and 50° and the seat cushion angle between 30° and 40°. When the angle of the backrest exceeds 65°, the pressure on the lower back exceeds the ideal range and increases the risk of thoracic vertebra injury, so simply increasing the backrest angle does not guarantee improved comfort; the comfort level is the highest when the backrest angle is rotated backward within the range of 0°–10° from the basic angle. Changes in thoracic and lumbar vertebral stress align closely with variations in spinal angles, and the lumbar spine angle is closely related to the lumbar disc stress. Spinal angle changes can serve as reliable indicators of stress conditions. Furthermore, the study establishes a correlation between spinal loading and surface pressure, highlighting the potential of body pressure distribution data to reflect spinal responses. The average pressure and contact area in the pressure distribution can be used to predict the maximum stress on the intervertebral disc. The maximum pressure in the pressure distribution can be used to predict the maximum strain of the intervertebral disc. In practical applications, the index of body pressure distribution can be used to predict the force on the spine. These results provide a foundation for optimizing seat angle design to enhance comfort while maintaining spinal health.

## Practitioner Summary

In order to find the most comfortable seat angle and reveal the relationship between spinal force and body pressure distribution, an FE model was developed to study the responses of the Chinese human body under different seat angles. The study concludes that increasing the backrest angle does not necessarily enhance comfort.

## Data Availability

The original contributions presented in the study are included in the article/supplementary material; further inquiries can be directed to the corresponding author.
